# Comparative data analsysis of two multi-drug resistant homoserine lactone and rhamnolipid producing *Pseudomonas aeruginosa* from diabetic foot infected patient

**DOI:** 10.1016/j.dib.2020.106071

**Published:** 2020-07-25

**Authors:** Prakhar Srivastava, Sankaranarayanan Gomathinayagam, Nalini Easwaran, Gowri Sankar, E. Padmavathi, Manoharan Shankar, Kodiveri M. Gothandam, Karthikeyan Sivashanmugam

**Affiliations:** School of Bio Sciences and Technology, Vellore Institute of Technology, Vellore 632014 India

**Keywords:** Diabetic foot ulcer, Biofilm, Quorum sensing, Homoserine lactone, Efflux pump

## Abstract

*Pseudomonas aeruginosa* generally forms strong biofilm during chronic condition of wound. The whole mechanism of biofilm formation works in tandem with quorum sensing circuit of the organism in order to produce virulence. Here we report the draft genome sequence of two diabetic foot ulcer *Pseudomonas aeruginosa* isolates (VIT PC 7 and VIT PC 9) displaying homoserine lactone, rhamnolipid producing, biofilm phenotype and antibiotic resistance genes related to carbapenem, aminoglycoside, beta- lactamase and tetracycline resistance. The whole genome sequencing library was prepared according to the Oxford Nanopore's SQK-LSK108 kit protocol on Oxford Nanopore's Minion platform. The 7.1 Mb and 6.3-Mb draft genome sequence with GC content of 65.8% and 66.4% respectively provides insight into their resistance mechanism and virulence factors.

**Specifications Table**

**Table:** 

**Subject**	Microbiology
**Specific subject area**	Immunology and Microbiology
**Type of data**	Whole genome sequence with gene annotation and comparative genomic of multi-drug resistant *Pseudomonas aeruginosa* strain VIT PC 7 and VIT PC 9.
**How data were acquired**	The whole genome sequencing library was prepared according to the Oxford Nano pore's SQK-LSK108 kit protocol on Oxford Nano pore's Minion platform. Base calling was done using guppy base caller v1.6.0, and assembly was performed *de novo* using *Flye* assembler version 2.6.
**Data format**	Raw analysed
**Parameters for data collection**	VIT PC 7 and VIT PC 9 isolates of *Pseudomonas aeruginosa* from diabetic foot ulcer patient's sample, were characterized for their biochemical, 16sRNA sequencing, antibiotic susceptibility (disk diffusion assay), biofilm assay and whole genome sequencing for collecting data.
**Description of data collection**	Sequencing library of *Pseudomonas aeruginosa* VIT PC 7 and VIT PC 9 was prepared and assembled using guppy base caller v1.6.0 and Flye assembler version 2.6. For these sequences, NCBI Prokaryotic Genome Annotation Pipeline version 4.2, Rapid Annotations using Subsystems Technology (RAST) server, ResFinder and anti-SMASH analysis tool were used for data collection.
**Data source location**	Institution: Vellore Institute of Technology, Vellore and Government Vellore Medical College (GVMC), Vellore
	City/Town/Region: Vellore, Tamil Nadu
	Country: India
**Data accessibility**	**Repository name:** NCBI (National centre for Biotechnology Information) GenBank Nucleotide database associated with Bio project number.
	**Data identification number:** accession numbers JAAGOT000000000.1 and CP048791.1 for P. aeruginosa VIT PC 7 and VIT PC 9, respectively, raw sequence reads have been deposited under accession numbers SAMN14054257 and SAMN14054838 for P. aeruginosa VIT PC 7 and VIT PC 9, respectively, Bio Project accession number: PRJNA605314 and PRJNA605318, respectively.
	Direct URL to assembled data:
	**For VIT PC 7**
	https://www.ncbi.nlm.nih.gov/nuccore/JAAGOT00000000.1
	https://www.ncbi.nlm.nih.gov/bioproject/PRJNA605314
	https://www.ncbi.nlm.nih.gov/biosample/SAMN14054257
	**For VIT PC 9**
	https://www.ncbi.nlm.nih.gov/nuccore/CP048791.1/
	https://www.ncbi.nlm.nih.gov/bioproject/?term=PRJNA605318
	https://www.ncbi.nlm.nih.gov/biosample/?term=SAMN14054838
	**Direct URL to raw data:**
	https://www.ncbi.nlm.nih.gov/sra/?term=PRJNA605314
	https://www.ncbi.nlm.nih.gov/sra/?term=PRJNA605318

**Value of the Data**

• The genome sequence represents a valuable resource for studies on the antibiotic resistance, pathogenicity analysis and expression of homoserine lactone from multiple drug resistant strain to enhance the understanding of *Pseudomonas aeruginosa* isolated from diabetic foot region.
• The data will give insight onto the resistance mechanism and genetic map of the isolate which will benefit patients, researchers and clinicians.
• This genome data might provide efficient information regarding the genetic makeup of organism and will pave way to design more specific drugs and therapeutic tools.
• Bacterial whole genome sequence (WGS) is a very productive and beneficial way in order to deduce the various complex mechanism of an isolate and also it helps in understanding the complex nature of microbes even if it is polymicrobial or monomicrobial infection.
• The presence of genes and other metabolic blocks shows the entire network of the proteins and genes, WGS can be used in identifying the potent therapeutic targets in order to generate efficient medicines related to genetic makeup of the organism.

## 1 Data description

**Fig 1:**
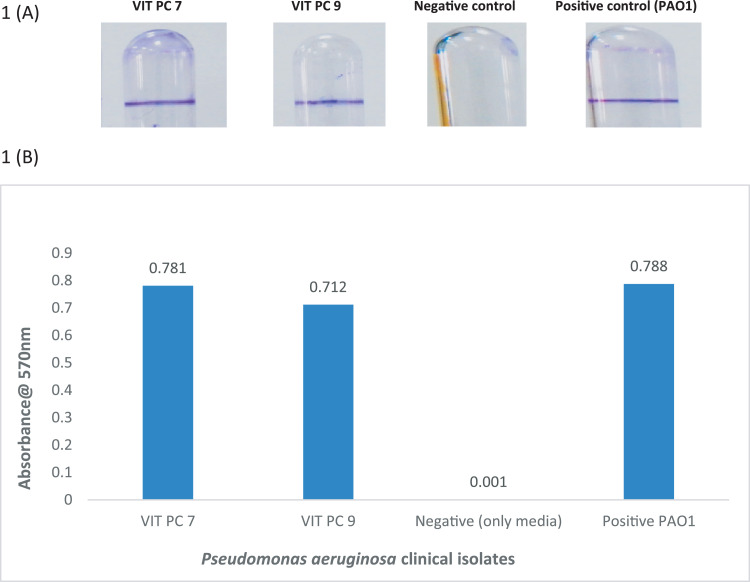
A-B. Crystal violet assay and quantification analysis of biofilm formation by *Pseudomonas aeruginosa* VIT PC 7, VIT PC 9, *Pseudomonas aeruginosa* PAO1(positive control) and negative control (only media).

*Pseudomonas aeruginosa* is generally an opportunistic pathogen [1], which have an active role in increasing the chronicity of the wound [2], *Pseudomonas aeruginosa* infection has been found in various level of skin infections [3], starting from subcutaneous to the deeper part of the wound [4], Polymicrobial infection during foot ulcers are prominent with *pseudomonas aeruginosa* infection [5], as *Pseudomonas aeruginosa*  have efficient tendency to form strong biofilm [6], biofilm formation and quorum sensing works in tandem in *Pseudomonas aeruginosa* complex genome structure [7] were observed using ResFinder (https://cge.cbs.dtu.dk/services/ResFinder/) **(as supplementary file 1 and 2.**), detected 4 genes coding for sulphonamide resistance [ sul1] for VIT PC 7,  3 genes coding for aminoglycoside resistance[ aph(3″)-Ib, aph(3″)-Ib, aph(3′)-VI, aph(6)-Id] for VIT PC 7 and aph(3′)-llb for VIT PC 9, two genes coding for Beta-lactam resistance [bla_OXA_-488, bla_PAO_] for VIT PC 7 and bla _OXA-396_ , bla _OXA-494_ and bla_PAO_ for VIT PC 9, quinolone resistance [crpP, qnrVC1] only found in VIT PC 7 and one gene coding fosfomycin resistance [fosA] in VIT PC 7 and VIT PC 9 respectively. Phenicol resistance [catB7] was found in both the isolates, Tetracycline resistance [tet(G)] was found only in VIT PC 7. Automated bioinformatics analysis tool (https://antismash.secondarymetabolites.org/#!/start) (**as supplementary file 3a,3b,4a and 4b.**) predicted 10 (VIT PC 7) and 11 (VIT PC 9) biosynthetic gene clusters coding for secondary metabolites, clusters for hserlactone (homoserine lactone), bacteriocin, phenazine, beta lactone, pyocyanin, pyochelin, pyrrolizixenamide and thanamycin presence in both the isolates, apart from these clusters pyoverdine was only present in VIT PC 9. The remaining clusters were predicted to encode N acetyl glutaminyl glutamine amide (NAGGN), Non-ribosomal peptide synthetase cluster (NRPS) and L-2-amino-4-methoxy-trans-3-butenoic acid compounds. Rapid Annotations Subsystems Technology [8] (https://rast.nmpdr.org/rast.cgi) identified genes associated with resistance to antibiotics and toxic compounds, including Mex AB, MexC and MexD efflux pump, copper homeostasis, cobalt-zinc-cadmium resistance, copper homeostasis: copper tolerance, fosfomycin resistance, beta-lactamase, efflux pump resistance, chromium compound resistance and resistance to fluoroquinolones, RAST analysis also showed the presence of C4-HSL and 3-oxo-C12-HSL (Las R and Rhl R transcriptional activator) respectively ( **as supplement 5 and 6**). Biofilm analysis for VIT PC 7 and VIT PC 9 was also performed using crystal violet assay and was quantified against negative and positive control (*Pseudomonas aeruginosa* PAO1) **(**Fig 1 **A-B).**

## 2 Experimental design, materials and methods

### 2.1 Collection of pseudomonas aeruginosa isolates

*Pseudomonas aeruginosa* strains were isolated from the pus region of the adult patients suffering from a chronic condition of diabetic foot ulcer at Government Vellore Medical College (GVMC), Vellore, Tamil Nadu. The procedure to collect and process the samples were approved by the Institutional Ethical Committee of GVMC. These pus samples were collected in swab tube and were given to department of microbiology (GVMC), for isolation of bacteria. Bacteria from the pus samples were grown on King's Medium B Base agar at 37 °C and identified as *Pseudomonas aeruginosa*; further, these isolates were confirmed as *Pseudomonas aeruginosa* by using 16sRNA sequencing, Isolates were then subjected to disk diffusion antibiotic sensitivity testing [9], five different classes of antibiotics (Piperacillin/Tazobactam, Gentamicin, ciprofloxacin, Ceftazidime and Meropenem), concentration 0f 100/10 µg for piperacillin/tazobactam, 30 µg for ceftazidime, 10 µg for meropenem, 10 µg for gentamicin and 5 µg for ciprofloxacin were used and the strains were found to be resistant against the isolate putting it in the criteria of drug-resistance (Table 1.).

**Table 1:** disk diffusion method using CLSI guidelines (PIP/TAZ-Piperacillin Tazobactam, CEF-Ceftazidime, MER-Meropenem, GEN-Gentamicin, CIP-Ciprofloxacin) (R-resistant; S- susceptible; I- Intermediate), Zone diameter interpretive criteria in mm according to CLSI guidelines (PIP/TAZ- ≥21 (S), 15–20 (I), ≤14 (R) ; CEF- ≥18 (S), 15–17 (I), ≤14 (R) ; MER- ≥19 (S), 16–18 (I), ≤15 (R) ; GEN- ≥15 (S), 13–14 (I), ≤12 (R) ; CIP- ≥21 (S), 16–20 (I), ≤15 (R)).

Isolates	PIP/TAZ	CEF	MER	GEN	CIP
VIT PC CL-7	12 mm (R)	17 mm (I)	12 mm (R)	≤12 mm (R)	8 mm (R)
VIT PC CL-9	14 mm (R)	<13 mm (R)	≤12 mm (R)	≤12 mm (R)	18 mm (I)

### 2.2 DNA extraction and quality control

The *Pseudomonas aeruginosa* isolates (VIT PC 7 and VIT PC 9) were cultured for 16 hrs at 30°C in cation adjusted Muller Hinton Broth (CAMHB) (Himedia Laboratories). The genomic DNA was extracted using Qiagen's Bacterial All Prep^Ⓡ^ DNA/RNA Protein isolation kit and the purity of the isolated DNA was estimated using Qubit 4.0 Fluor meter with 1x dsDNA HS assay kit.

### 2.3 Library preparation and sequencing

Sequencing library was prepared according to the Oxford Nano pore's SQK-LSK108 kit protocol, and was loaded onto a flow cell (R9.4 chemistry) and ran for 12 hours on Oxford Nano pore's Minion platform. Base calling was done using Guppy base caller v1.6.0, and assembly was performed *de novo* using *Flye* assembler version 2.6 [10]. It yielded a draft genome for strain VIT PC7 with a genome size of 7116,506 bp in 6 contigs and strain VIT PC9 has a genome size of 6367,369 bp, with GC content of 65.8% and 66.4% respectively. The sequences were annotated using NCBI Prokaryotic Genome Annotation Pipeline version 4.2 and analyzed by the Rapid Annotations using Subsystems Technology (RAST) server. The annotation process detected a total of 6818 and 5916 genes, respectively, of which 4556 and 3900 are coding sequences (CDSs). These Coding sequences are sorted into 391 and 385 subsystems respectively. Out of the above mentioned CDSs 2182 and 1937 are pseudogenes (which is likely due to the intrinsic errors in the adapted sequencing technology), and 80 RNAs respectively, including 64 tRNA genes 4 rRNA genes, and 4 noncoding RNAs (ncRNAs) respectively.

## Ethics statement

The Institutional Ethical Committee approval certificate was obtained from Government Vellore Medical Hospital (GVMC), Vellore, Tamil Nadu for collection of pus sample from diabetic foot ulcer patients.       The Institutional Ethical committee (IEC) meeting was held on 06–10–2016 at conference hall, (GVMC), Vellore. The members of the committee, secretary, convenor and the president approved the proposed work entitled “In vitro and In vivo studies of inhibitory effect of *Bacillus* derived N-acyl Homoserine Lactonase degrading enzyme (Aiia) on *Pseudomonas aeruginosa* in Diabetic Foot Ulcer”.

## Declaration of Competing Interest

The authors declare that they have no known competing financial interests or personal relationships which have, or could be perceived to have, influenced the work reported in this article.

## Appendix Supplementary materials

Image, application 1

Image, application 2

Image, application 3

Image, application 4

Image, application 5

Image, application 6

Image, application 7

Image, application 8

Image, application 9

## References

[1] Valot B., Guyeux C., Rolland J.Y., Mazouzi K., Bertrand X., Hocquet D. (2015). What it takes to be a Pseudomonas aeruginosa? The core genome of the opportunistic pathogen updated. PLoS ONE.

[2] Georgescu M., Gheorghe I., Curutiu C., Lazar V., Bleotu C., Chifiriuc M.C. (2016). Virulence and resistance features of Pseudomonas aeruginosa strains isolated from chronic leg ulcers. BMC Infect. Dis..

[3] Nguyen L., Garcia J., Gruenberg K., MacDougall C. (2018). Multidrug-resistant pseudomonas infections: shard to treat, but hope on the horizon?. Curr. Infect. Dis. Rep..

[4] Trøstrup H., Lerche C.J., Christophersen L.J., Thomsen K., Jensen P.Ø., Hougen H.P., Høiby N., Moser C. (2017). Chronic Pseudomonas aeruginosa biofilm infection impairs murine S100A8/A9 and neutrophil effector cytokines—Implications for delayed wound closure?. Pathog. Dis..

[5] Nguyen A.T., Oglesby-Sherrouse A.G. (2016). Interactions between Pseudomonas aeruginosa and Staphylococcus aureus during co-cultivations and polymicrobial infections. Appl. Microbiol. Biotechnol..

[6] Percival S.L., Malone M., Mayer D., Salisbury A.M., Schultz G. (2018). Role of anaerobes in polymicrobial communities and biofilms complicating diabetic foot ulcers. Int. Wound J..

[7] Lin J., Cheng J. (2019). Quorum sensing in pseudomonas aeruginosa and its relationship to biofilm development. Int. Biofilm Eng. Am. Chem. Soc..

[8] Aziz R.K., Bartels D., Best A.A., DeJongh M., Disz T., Edwards R.A., Formsma K., Gerdes S., Glass E.M., Kubal M., Meyer F. (2018). The RAST Server: rapid annotations using subsystems technology. BMC Genom..

[9] Clinical and Laboratory Standards Institute. M100-S25 performance standards for antimicrobial susceptibility testing; twenty-fifth informational supplement, 2015.

[10] Kolmogorov M., Yuan J., Lin Y., Pevzner P.A. (2019). Assembly of long, error-prone reads using repeat graphs. Nat. Biotechnol..

